# A novel scoring system for predicting the neurologic prognosis prior to the initiation of induced hypothermia in cases of post-cardiac arrest syndrome: the CAST score

**DOI:** 10.1186/s13049-017-0392-y

**Published:** 2017-05-10

**Authors:** Mitsuaki Nishikimi, Naoyuki Matsuda, Kota Matsui, Kunihiko Takahashi, Tadashi Ejima, Keibun Liu, Takayuki Ogura, Michiko Higashi, Hitoshi Umino, Go Makishi, Atsushi Numaguchi, Satoru Matsushima, Hideki Tokuyama, Mitsunobu Nakamura, Shigeyuki Matsui

**Affiliations:** 1 Department of Emergency and Critical Care, Nagoya University Graduate School of Medicine, Tsurumai-cho 65, Syowa-ku, Nagoya, Aichi 4668560 Japan; 2 Department of Biostatistics, Nagoya University Graduate School of Medicine, Tsurumai-cho 65, Syowa-ku, Nagoya, Aichi 4668560 Japan; 3 Advanced Medical Emergency Department and Critical Care Center, Japan Red Cross Maebashi Hospital, Asahi-cho 3-21-36, Maebashi, Gunma 3710014 Japan; 4 Department of Emergency and Critical Care, Cyutouen General Medical Center, Shobugaike 1-1, Kakegawa, Shizuoka 4368555 Japan

**Keywords:** Post-cardiac arrest syndrome, Neurological prognosis, Induced hypothermia, CAST score

## Abstract

**Background:**

The aim of this study was to develop a scoring system for identifying the post-cardiac arrest syndrome (PCAS) patients with a good potential for recovery prior to the initiation of induced therapeutic hypothermia.

**Methods:**

A multi-center, retrospective, observational study was performed. Data of a total of 151 consecutive adults who underwent induced hypothermia after cardiac arrest (77 learning cases from two hospitals and 74 validation cases from two other hospitals) were analyzed.

**Results:**

In the learning set, 8 factors (initial rhythm, witnessed status and time until return of spontaneous circulation, pH, serum lactate, motor score according to the Glasgow Coma Scale (GCS), gray matter attenuation to white matter attenuation ratio (GWR), serum albumin, and hemoglobin) were found to be strongly correlated with the neurological outcomes. A tentative scoring system was created from the learning data using these factors, and the predictive accuracy (sensitivity and specificity) was evaluated in terms of both internal validation (0.85 and 0.84) and external validation (cutoff 50%: 0.95 and 0.90, 30%: 0.87 and 0.98, 15%: 0.67 and 1.00). Finally, using all the data, we established a post-Cardiac Arrest Syndrome for induced Therapeutic hypothermia (CAST) score to predict the neurologic prognosis prior to initiation of induced hypothermia.

**Conclusions:**

The CAST score was developed to predict the neurological outcomes of PCAS patients treated by induced hypothermia. The likelihood of good recovery at 30 days was extremely low in PCAS patients with a CAST score of ≤15%. Prospective validation of the score is needed in the future.

**Electronic supplementary material:**

The online version of this article (doi:10.1186/s13049-017-0392-y) contains supplementary material, which is available to authorized users.

## Background

Care of patients with post-cardiac arrest syndrome (PCAS) is mainly aimed at improving the neurological prognosis of the patients after the return of spontaneous circulation (ROSC) [1, 2]. To improve the outcomes of PCAS patients, many hospitals perform undertake induced therapeutic hypothermia, based on the results of previous randomized controlled trials [3–7]. Prediction of the prognosis of PCAS patients at the time of their arrival in the Emergency Room before the initiation of induced hypothermia will be useful for stratifying patients for precise clinical research, as well as for providing a baseline estimation of their prognosis.

Some studies have examined several factors that are quantifiable before the initiation of induced hypothermia to determine their relationships to the neurological outcomes in PCAS patients. For example, the duration of the resuscitation effort has been shown to be correlated with a good functional outcome in patients with PCAS [8, 9]. Other studies have revealed correlations between the pH [10], serum lactate [11, 12] and Glasgow Coma Scale (GCS) score [13, 14] with the neurological prognosis in PCAS patients. However, none of these factors has been found to be by itself capable of satisfactorily separating patients with a good outcome from those with a poor outcome, suggesting that establishment of a “suitable scale” based on a combination of prognostic factors might be useful [15, 16]. The aim of the present study was to develop a prognostic scoring system for predicting the neurologic prognosis in PCAS patients treated with induced hypothermia based on the results of examinations carried out prior to the initiation of induced hypothermia. A summary of this study was previously reported in a letter format [17].

## Methods

### Study design

We conducted a multi-center, retrospective, observational study examining adult patients who were treated with induced hypothermia after experiencing cardiac arrest. We retrospectively reviewed the clinical management charts of the eligible patients who were admitted to our hospitals during a period spanning the last 3-5 years: 54 patients treated at Nagoya University Hospital between April 2011 and March 2016, 23 patients treated at Chutouen General Medical Center between April 2013 and March 2016, 64 patients treated at Japan Red Cross Maebashi Hospital between April 2011 and March 2016, and 10 patients treated at Komaki City General Hospital between April 2012 and March 2016. Eligible patients were all who were treated with induced hypothermia after experiencing cardiac arrest (induced hypothermia was considered for cardiac arrest patients who were in a coma (GCS ≤ 8) after ROSC without remarkable hemodynamic instability or a “Do Not Attempt to Resuscitation” directive.). They were excluded if they were traumatic cardiac arrest patients, or pediatric patients (age < 18 years), or did not have lived independently prior to experiencing cardiac arrest. For the purpose of developing and validating the prognostic scoring system, we divided all 151 patients into a learning set (77 cases treated at Nagoya University Hospital or Chutouen General Medical Center) and a validation set (74 cases treated at Komaki City General Hospital or Japan Red Cross Maebashi Hospital). These sets were created with the aim of developing and validating a scoring system for a broad population of patients from city hospitals and general medical centers in the countryside, so each set contained one hospital located in a city and one located in a countryside. This study was approved by the research ethics boards of Nagoya University Hospital, Chutouen General Medical Center, Komaki City General Hospital and Japan Red Cross Maebashi Hospital.

### Participating hospitals

The four participating hospitals are tertiary emergency medical centers (Japanese centers for emergency patients with serious or life-threatening conditions): Nagoya University Hospital is an academic hospital, while Chutouen General Medical Center, Japan Red Cross Maebashi Hospital, and Komaki City General Hospital are general hospitals. Nagoya University Hospital and Japan Red Cross Maebashi Hospital are both located in cities and have 1,035 and 592 beds each, including 26 and 12 ICU beds, respectively; these hospitals respectively treat about 12,000 and 20,000 emergency patients each year. Chutouen General Medical Center and Komaki City General Hospital are both located in the countryside and have 500 and 558 beds each, including 10 and 30 ICU/CCU beds; these hospitals treat about 20,000 and 30,000 emergency patients per year.

### Dataset

Data was collected retrospectively from electronic chart reviews, including the clinical histories (age, sex, situation surrounding the cardiac arrest), cardiac rhythms, physical examinations performed upon the patient’s arrival in the Emergency Room (GCS, mydriasis), results of blood examinations (C-reactive protein [CRP], albumin [Alb], hemoglobin [Hb], glucose, creatinine, pH, lactate), cranial CT scan images, and clinical courses after admission. Scoring variable candidates were selected from among parameters measurable before admission to the ICU and the initiation of induced hypothermia.

**Fig. 1:**
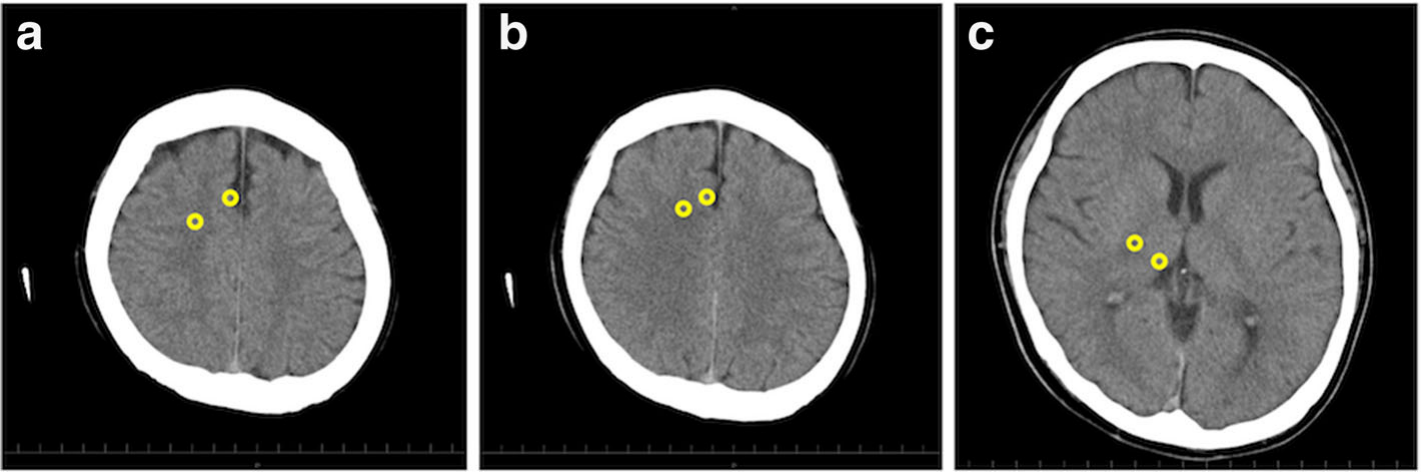
Three imaging slices used to calculate the gray matter attenuation to white matter attenuation ratio. Left, high convexity level (**a**). Middle, centrum semiovale level (**b**). Right, basal ganglia level (**c**). In each slice, the Hounsfield units (HU) were measured within circle 1 on the gray matter and within circle 2 on the white matter. Then, each HU value was then calculated based on the average of the values for the right and left brains. The slice thickness was 5 mm, and the circle size was 10 mm^2^

The gray matter attenuation to white matter attenuation ratio (GWR) was measured using the method described in Torbey’s report [18]. CT scan images were obtained using an Aquilion64 (TOSHIBA) or SOMATOM Definition Flash (SIEMENS) within 6 h after the patient’s cardiac arrest event. As shown in Fig. 1, the intensities of circular areas of interest (about 10 mm^2^) were measured for both the gray matter and the white matter on three axial slices (5-mm slice thickness) at a basal ganglia level, a centrum semiovale level, and a high convexity level. Then, the GWR was calculated as shown below [19]:

$$ \mathrm{G}\mathrm{W}\mathrm{R}=\left(\left[\mathrm{Th}/\mathrm{PIC}\right]+\left[\mathrm{MC}1/\mathrm{MWM}1\right]+\left[\mathrm{MC}2/\mathrm{MWM}2\right]\right)/3 $$

where Th represents the thalamus, MC1 represents the medial cortex at the centrum semiovale, MC2 represents the medial cortex at the high convexity level, PIC represents the posterior limb of the internal capsule, MWM1 represents the medial white matter at the centrum semiovale, and MWM2 represents the medial white matter at the high convexity level. Each value was the average of the right and left hemisphere values.

Before developing the scoring system for the learning set, we conducted some unit imputations because the baseline data involved some missing values. The mean value was used for the missing values of continuous variables, while 0 was used for the missing values of binary variables. Of note, we confirmed that the accuracy of the prediction did not change even if a value of 1 was used for the missing values.

### Protocol for therapeutic hypothermia

Induced hypothermia was performed for eligible patients according to each hospital’s protocol. Induced hypothermia was considered for cardiac arrest patients who were in a coma (GCS ≤ 8) after ROSC without remarkable hemodynamic instability or a “Do Not Attempt to Resuscitation” directive. At Nagoya University Hospital, Chutouen General Medical Center, and Komaki City General Hospital, a temperature of 34 °C was targeted by the infusion of cold fluids in combination with surface cooling, an ice pack and cold blanket, or a surface cooling device with a computerized automatic temperature control (Arctic Sun 2000 TTM; Bard Medical Louisville, CO). After the targeted temperature had been maintained for 24 h, rewarming to 36 °C was performed at a rate of 0.2 °C/4 h. Propofol, dexmedetomidine, fentanyl and rocuronium were used for sedation, analgesia, and muscle relaxation according to individual clinician preferences. At the Japan Red Cross Maebashi Hospital, the target temperature was mostly 34 °C, but the target was changed to 35 °C or 36 °C if the patient experienced hemodynamic instability. Induced hypothermia was performed using a surface-cooling device with a computerized automatic temperature control (Arctic Sun 2000 TTM; Bard Medical Louisville, CO). After the targeted temperature had been maintained for 24 h, rewarming to normothermia was performed at a rate of 1 °C/24 h, stopping at 36 °C. Propofol, dexmedetomidine, midazolam, fentanyl and rocuronium were used for sedation, analgesia, and muscle relaxation according to individual clinician preferences. At all the participating hospitals, the ventilator settings, fluid infusion, and doses of vasopressors, sedatives, and analgesics were adjusted so that the mean arterial pressure, pCO_2_, and urine output were ≥80 mmHg, 35-45 mmHg, and ≥0.5 mL/kg/h, respectively, to maintain cerebral perfusion.

### Neurological outcome

We used the Cerebral Performance Categories (CPC) at 30 days to estimate the neurological outcomes as follows: CPC 1, full recovery; CPC 2, moderate disability; CPC 3, severe disability; CPC 4, coma or vegetative state; and CPC 5, died [20]. We calculated the CPC score at 30 days by reviewing the electronic charts or interviewing the patient’s family and the institutions where the patients were admitted at 30 days. The categories were grouped into either a good outcome (1-2) or a poor outcome (3-5) [20].

### Sample size for external validation

We determined the sample size for the external validation set so as to ensure a high precision in estimating the proportion of correct classification (PCC). Specifically, we set the target value of PCC as 85%, which is the point estimate for the classifier based on the logistic regression obtained in the internal validation. We required that the width of a two-sided confidence interval with a 95% confidence level based on a normal approximation be less than 10%. In this manner, we determined that at least 51 patients were needed for the external validation.

### Statistical analysis

During the development of the prognostic scoring system, we first identified a set of prognostic factors that could be used to predict the clinical outcome, that were both clinically important and routinely measurable in the Emergency Room, and that also exhibited some correlation with the outcome variable in the learning set. In developing classifiers using the resulting set of prognostic factors, we applied the standard logistic regression and decision tree algorithms to the learning set. For the internal validation of these classifiers, we conducted a 10-fold cross-validation using the learning set to estimate the indices of the predictive accuracy, including the proportion of correct classification, specificity, and sensitivity. Here, specificity measures the proportion of patients with poor outcomes who were correctly identified as such. We repeated the cross-validation analysis 50 times with different random sample splits in the learning set to obtain stable estimates of these indices. We then applied the prediction algorithm with a higher accuracy in the internal validation to all the patients in the learning set to obtain a classifier for the external validation. In the cross-validation, we also identified the most appropriate number of the variables for the scoring system by comparing the predictive accuracies between different numbers of variables. In the external validation, we estimated each 95% confidence interval (95% CI) using an exact method based on the beta distribution (a normal approximation was not used). Finally, we applied the prediction algorithm to the entire 151 patients to create a novel scoring system for use with future patients. R software was used for all the statistical analyses. We used the “glmnet” package for logistic regression (http://www.jstatsoft.org/v33/i01/) and the “rpart” package for the decision tree (http://CRAN.R-project.org/package=rpart). In “glmnet” package, the logistic regression algorithm allows a “good prognosis” to be identified if the posterior probability of a good prognosis is greater than 50%; otherwise, a “poor prognosis” is identified.

## Results

**Fig. 2:**
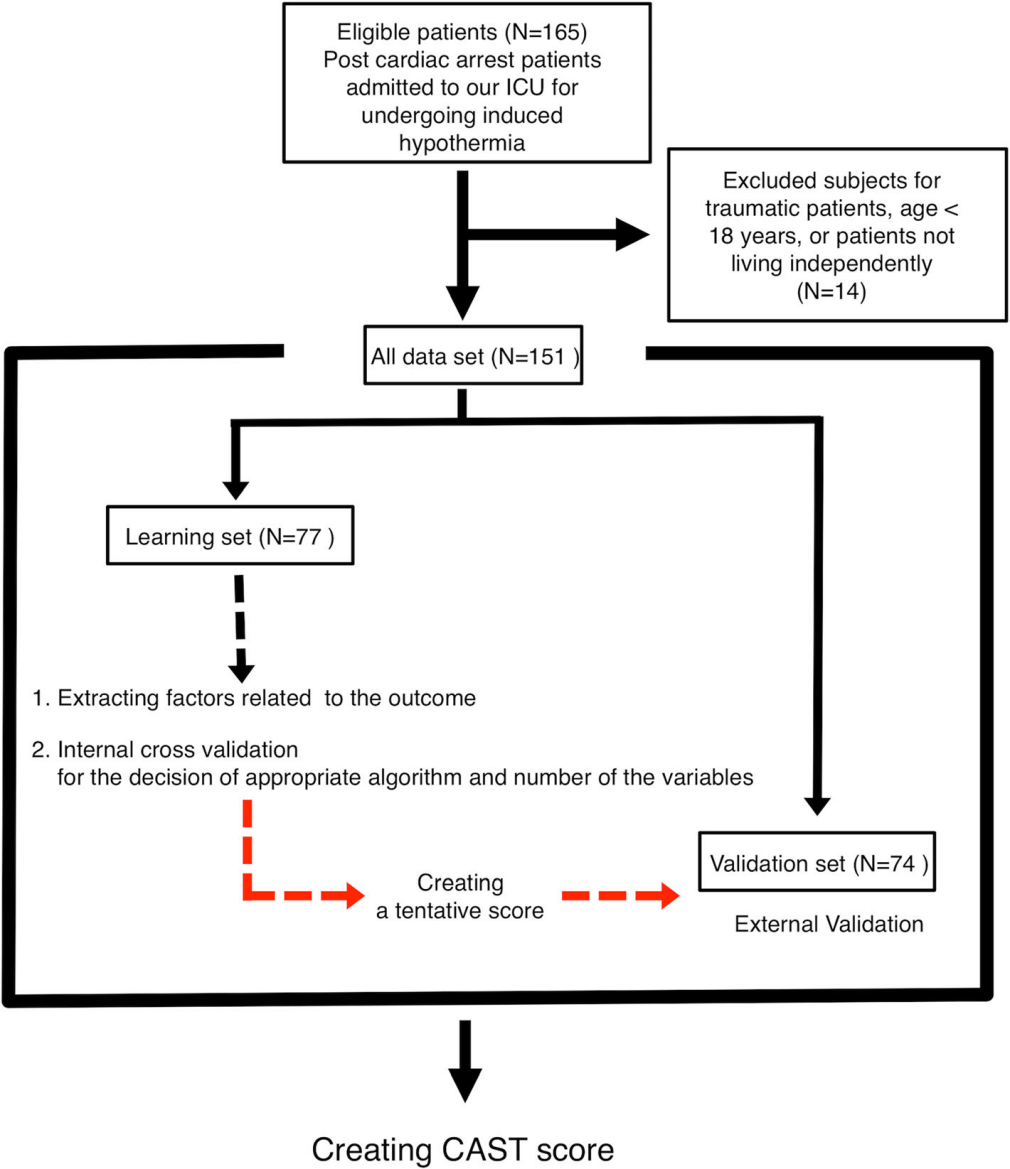
Flow chart illustrating the subject enrollment and development of the score

A flow chart illustrating the subject enrollment and development of the score is shown in Fig. 2. A total of 151 consecutive adults were divided into learning set in two hospitals and validation set in other two hospitals. From the learning set, we extracted several factors for developing the scoring system and performed an internal cross validation in order to decide the optimum statistical algorithm and number of variables. A tentative scoring system was created from the data, and the predictive accuracy of the scoring system was examined by external validation. Finally, using all of the data, we created the post-Cardiac Arrest Syndrome for induced Therapeutic hypothermia (CAST) score to predict the neurological prognosis in PCAS patients prior to the initiation of induced hypothermia.

The baseline characteristics of the patients in the learning and validation sets are summarized in Table 1. The correlations between the baseline variables and the patient outcomes in the learning set are shown in Table 2. Candidate variables for inclusion in the scoring system were selected from among the variables that were examined before the patients were admitted to the ICU for the initiation of induced hypothermia.

**Table 1:** Baseline Characteristics of the Learning and Validation Data Sets

Variable	Learning data set		Validation data set	
	Nagoya *n* = 54	Chutouen *n* = 23	Komaki *n* = 10	Maebashi *n* = 64
Demographics				
Age, yr	62.5 (52.0-70.0)	67.0 (55.5-75.0)	47.0 (27.0-51.0)	61.0 (52.0-72.0)
Length of stay in hospital, d	27.5 (17.0-52.3)	24.0 (9.0-52.0)	11.5 (5.8-52.5)	30.5 (18.0-67.5)
Condition of cardiac arrest				
Witness, n (%)	43 (79.6)	18 (78.3)	8 (80.0)	51 (79.7)
Bystander, n (%)	28 (51.9)	15 (65.2)	4 (40.0)	36 (56.3)
Initial rhythm, shockable, n (%)	30 (55.6)	8 (36.4)	4 (40.0)	45 (70.3)
Duration of resuscitation effort, min	20.0 (13.0-30.0)	20.5 (11.0-39.3)	24.0 (12.5-33.3)	15.0 (9.0-30.0)
Time to reach target temperature after cardiac arrest, hour	5.0 (3.5-6.0)	4.8 (4.0-6.8)	6.0 (4.0-8.0)	5.0 (3.0-8.0)
Outcome				
Good (CPC ≤ 2), n, (%)	23 (42.6)	11 (47.8)	3 (30.0)	27 (42.2)
Poor (CPC ≥ 3), n, (%)	31 (57.4)	12 (52.2)	7 (70.0)	37 (57.8)

Data are presented as the median and interquartile ranges (25–75% percentile) or as absolute frequencies with percentages. *Nagoya* Nagoya University Hospital, *Chutouen* Chutouen General Medical Center, *Komaki* Komaki City General Hospital, *Maebashi* Japan Red Cross Maebashi Hospital

**Table 2:** Correlation Coefficients and *P* Values between Each Variable and Patient Outcomes in the Learning Data Set

Variable	Good (CPC ≤ 2) *n* = 34	Poor (CPC ≥ 3) *n* = 43	*r*	*P*
Age	62.3 ± 13.7	60.1 ± 17.2	0.07	0.550
Witness, n (%)	22 (91.7)	21 (65.6)		
Time until ROSC, min	20.1 ± 17.6	28.9 ± 14.8		
Witness/Time until ROSC			- 0.48	<0.001
Bystander, n (%)	19 (55.9)	24 (55.8)	0.00	0.995
Initial rhythm, shockable, n (%)^a^	24 (72.7)	14 (32.6)	0.40	<0.001
GCS, M ≥ 2, n (%)^b, h^	29 (87.9)	13 (31.7)	0.56	<0.001
Mydriasis^c, h^	7 (21.2)	19 (45.2)	- 0.25	0.030
pH^d, i^	7.23 ± 0.13	6.98 ± 0.21	0.58	<0.001
Lactate (mmol/dL)^e, i^	8.0 ± 4.0	11.0 ± 4.2	- 0.35	0.003
WBC (10^3^/ L)^i^	10.3 ± 3.4	11.4 ± 4.8	- 0.13	0.263
CRP (mg/dL)^f, i^	0.20 ± 0.30	1.83 ± 4.24	- 0.25	0.031
Glucose (mg/dL)^i^	253 ± 89	266 ± 119	- 0.06	0.601
Creatinine (mg/dL)^i^	2.1 ± 3.2	2.3 ± 3.3	-0.03	0.769
Albumin (g/dL)^i^	3.9 ± 0.4	3.2 ± 0.7	0.48	<0.001
Hemoglobin (g/dL)^i^	14.1 ± 1.8	12.3 ± 2.5	0.37	<0.001
GWR^g, j^	1.26 ± 0.05	1.21 ± 0.08	0.33	0.005

Data are presented as mean ± standard deviation or as absolute frequencies with percentages

Missing data; ^a^ *n* = 1 and 0; ^b^ *n* = 1 and 2; ^c^ *n* = 1 and 1; ^d^ *n* = 1 and 3; ^e^ *n* = 1 and 6; ^f^ *n* = 1 and 2; ^g^ *n* = 3 and 5

^h^These data was obtained just after return of spontaneous circulation

^i^These data was obtained about 15 min before and after return of spontaneous circulation

^j^The CT was performed within 6 h after the patient had the return of his or her spontaneous circulation

*r* correlation ratio, *ROSC* return of spontaneous circulation, *GCS* Glasgow Coma Scale, *CRP* C-reactive protein, *GWR* gray matter attenuation to white matter attenuation ratio

From among the variables that were considered, we selected 8 factors (initial rhythm, witnessed status and time until ROSC, GCS-M score, pH, serum lactate, Alb, Hb and GWR) that showed significant correlations (*P* <0.01) with the patient outcomes. For convenience, while using the variables to create the scoring system, we converted the continuous variables into categorical variables in such a manner that higher scores implied poorer outcomes (Table 3).

**Table 3:** Categorical Classification of Each Variable

Score	0	1	2	3
Initial Rhythm (X1)	Shockable	Non Shockable		
Witness/ROSC time (*X*2)	<20 min	20 min ≤	No Witness	
pH (X3)	≥7.31	7.30–7.16	7.15–7.01	7.00 ≥
Lactate (X4)	≤5.0	5.1–10.0	10.1–14.0	14.1 ≤
GCS M (X5)	≥2	1		
GWR (X6)	≥1.201	1.200–1.151	1.150 ≥	
Alb (X7)	≥3.6	3.5–3.1	3.0 ≥	
Hb (X8)	≥13.1	13.0–11.1	11.0 ≥	

The cross-validated predictive accuracies (sensitivity, specificity, percentage of correct classifications) of the tentative scoring system created using the learning set were 0.85, 0.84 and 0.85 for the logistic regression algorithm, and 0.82, 0.73, and 0.78 for the decision-tree algorithm, respectively We used the logistic regression algorithm to construct the scoring system, because it yielded more accurate values for the sensitivity, specificity, and percentage of correct classification in the internal cross-validation, as compared to the decision-tree algorithm. The most appropriate number of variables was also confirmed using internal cross-validation. We compared the predictive accuracies of three scoring systems created with the logistic regression algorithm using different numbers of variables (6, 8, and 11 variables, which showed correlations with the outcomes at *P* <0.001, <0.01 and <0.05, respectively). The predictive accuracies of the three scoring systems were (0.86, 0.82, 0.84), (0.85, 0.84, 0.84), and (0.84, 0.83, 0.83), respectively. We decided to create the scoring system using 8 variables, because the highest specificity was obtained with this number of variables (specificity was prioritized because ethically, it may be more acceptable to misjudge a patient as being likely to have a good outcome, even if they actually have a poor outcome, than to misjudge a patient as being likely to have a poor outcome when they actually have a good outcome). An additional figure file illustrates this in greater detail [see Additional file 1].

Next, the 8-variable tentative scoring system that was developed from the learning set using the logistic regression algorithm was externally validated using a validation set consisting of 74 cases. For establishing the classification, we set 50, 30, and 15% as the cutoff values; for example, a 50% cutoff value meant that a “good prognosis” was identified if the probability of a good prognosis according to the score was greater than 50%; otherwise, a “poor prognosis” was identified. Based on these cutoff values, the sensitivity and specificity estimated using the validation set were (50%: 0.95 (95% CI, 0.82–0.99), 0.90 (0.77–0.99)), (30%: 0.87 (0.69–0.96), 0.98 (0.88–1.00)) and (15%: 0.67 (0.47–0.83), 1.00 (0.92–1.00)), respectively. Then, we plotted the receiver operator characteristic (ROC) curve and found an area under the curve (AUC) of 0.97 [see Additional file 2].

**Fig. 3:**
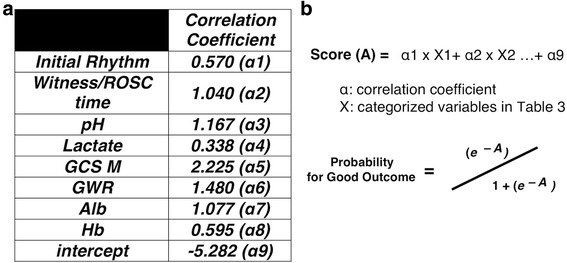
Calculation used for determining the post-Cardiac Arrest Syndrome for induced Therapeutic hypothermia (CAST) score. Using the correlation coefficients from all the data (**a**), the resultant scores and the probability of a good outcome were calculated (**b**)

Since the results of evaluation of the predictive accuracy of the scoring system by both internal and external validations implied that the process of creating the scoring system was appropriate, we finally developed a novel scoring system to predict the neurological outcomes of PCAS patients prior to their undergoing induced hypothermia (the post-Cardiac Arrest Syndrome for induced Therapeutic hypothermia (CAST) score)*,* by applying the logistic regression algorithm to all the 151 patients (including both the learning and validation sets; Fig. 3).

## Discussion

The considerable advances in neurological critical care medicine, including the use of induced hypothermia, have led to remarkable improvements in the neurological prognosis of PCAS patients. Such critical care treatments are, however, expensive and time-consuming (for example, the average total cost of admission of a PCAS patient treated by induced hypothermia at our hospital in 2015 was about $49,410 USD). Accurate prediction of the neurological outcomes before the initiation of induced hypothermia will help us to carefully consider the indications for these intensive and expensive therapies.

Although a few previous studies have attempted to establish a scoring system to estimate the neurological outcomes of PCAS patients at the time of their arrival in the Emergency Room, none of these studies took into account whether the patients included in the study had undergone/not undergone induced hypothermia [10, 21–24]. Today, many ICU doctors employ induced hypothermia in an attempt to obtain good recovery in patients in whom intensive treatment is indicated. It is noteworthy that the CAST score in this study was created using only data from PCAS patients who had undergone induced hypothermia, and not from all PCAS patients. Moreover, the factors included in the scoring systems attempted in these previous studies were mostly limited to clinical history items, such as the time until ROSC, the initial rhythm, and the witness status; no blood examination, physical examination or imaging findings were included. For the present study, we collected data from the patients’ clinical history, blood examination, physical examination and imaging findings, all of which were available prior to the initiation of induced hypothermia, and some of these parameters actually showed strong correlations with the patient outcomes. Using these findings, we created the CAST score to predict the neurological prognosis of PCAS patients even prior to the initiation of induced hypothermia.

When creating a scoring system, the convenience of the calculation should be emphasized, and until now, a simple decision-tree algorithm [22] or scoring system utilizing a simplified odds ratio [25] had been considered to be optimal. Today, however, electronic devices allow more complex formulae to be used, and utilize statistical algorithms with more logical and statistically verified backgrounds, enabling the creation of more accurate, and possibly more complex, scoring systems. In the present study, we judged that a logistic regression algorithm was optimal for establishing the CAST score system based on the results of an internal validation. To calculate the score more easily, we developed application tools for calculating the CAST score as iOS applications for the iPad [26] and iPhone [27].

Studies on predictive scores, while being extremely interesting, can also be contentious [28]. Predictive scores should be carefully considered, since they only show the probability of outcome in a general population, and not the precise probability for an individual patient. Although predictive scores can be useful for guiding decision-making and risk assessments for individual patients [28], their results are not absolute. Of course, the final therapeutic strategy should be decided based not only on the results of the scoring system, but also on different factors, such as the results of discussions with family members, the patient’s own wishes, societal considerations, etc. Most importantly, a patient’s exact neurological prognosis cannot be predicted without decisive examinations, such as an electroencephalogram or the long-term observation.

The predictive accuracy of the CAST score is limited, because it was created using retrospective data, even though its generalizability is likely to be high, since it was developed using data from multiple centers. We have to take into account that there were some missing values, for which we conducted some unit imputations. Moreover, it is possible that minor differences between hospital protocols (such as the kinds of sedatives or methods used for inducing and maintaining induced hypothermia) could have had some influence on the patient outcomes. The differences in the baseline characteristics of the patients among the participating hospitals can also not be ignored, because of the limited sample size of the study. It would be interest to conduct the further validation of the score using data from more participating hospitals. The endpoint used in this study was the outcome at 30 days. Although the outcome at 30 days has been used in a few other studies that have attempted to establish a predictive score for cardiac arrest patients [10, 29], it may be better to set longer-term end points, such as outcome at 90 days, for more accurate prediction of the future clinical course [30]. Prospective validation of the CAST score and a study to examine the usefulness of this score for predicting the long-term prognosis of PCAS patients are warranted.

## Conclusions

The CAST score was developed to predict the potential neurological outcomes of PCAS patients treated by induced hypothermia. According to our results, in PCAS patients with a CAST score of ≤15%, the likelihood of a good recovery at 30 days is extremely low. Prospective validation of the CAST score is needed in the future.

## Additional files

Additional file 1: Comparison of tentative scoring systems created using different algorithms and using different numbers of variables. The logistic regression algorithm yielded a higher sensitivity, specificity, and percentage of correct classification in the cross-validation study, than the decision-tree algorithm (A). The predictive accuracies of three scoring systems created using 6, 8, and 11 variables are shown (*P* values of 0.001, 0.01, and 0.05, respectively) (B). *Logistic* logistic regression algorithm, *Dec. tree* decision-tree algorithm, *Se* sensitivity, *Sp* specificity, *PCC* percentage of correct classification. (TIFF 16702 kb)

Additional file 2: Results of external validation of the tentative scoring system. The sensitivity, specificity, and percentage of correct classification for the external validation are shown (A). In these predictive accuracies, the cutoff values for discrimination between a poor and good prognosis was 50% (a “good prognosis” was identified if the probability of a good prognosis on the score was greater than 50%; otherwise, a “poor prognosis” was identified.), 30%, and 15%. The area under the receiver operating characteristic curve of the tentative score for which the logistic regression algorithm was applied with the different cutoff values (B). *ROC* receive operator characteristic. (TIFF 9281 kb)

## Abbreviations

PCAS: Post-cardiac arrest syndrome
GCS: Glasgow coma scale
GWR: Gray matter attenuation to white matter attenuation ratio
CAST score: Post-cardiac arrest syndrome for induced therapeutic hypothermia score
ROSC: Return of spontaneous circulation
ICU: Intensive care unit
CRP: C-reactive protein
Alb: Albumin
Hb: Hemoglobin
CPC: Cerebral performance categories
PCC: Proportion of correct classification
95% CI: 95% confidence interval
ROC: Receiver operator characteristic
AUC: Area under the curve
Nagoya: Nagoya University Hospital
Chutouen: Chutouen General Medical Center
Komaki: Komaki City General Hospital
Maebashi: Japan Red Cross Maebashi Hospital
r: Correlation ratio
Logistic: Logistic regression algorithm
Dec. tree: Decision tree algorithm
Se: Sensitivity
Sp: Specificity

## References

[1] Nolan JP, Soar J, Cariou A, Cronberg T, Moulaert VR, Deakin CD (2015). European resuscitation council and European society of intensive care medicine 2015 guidelines for post-resuscitation care. Intensive Care Med.

[2] Peberdy MA, Callaway CW, Neumar RW, Geocadin RG, Zimmerman JL, Donnino M (2010). Part 9: post-cardiac arrest care: 2010 American heart association guidelines for cardiopulmonary resuscitation and emergency cardiovascular care. Circulation.

[3] Hypothermia after Cardiac Arrest Study G (2002). Mild therapeutic hypothermia to improve the neurologic outcome after cardiac arrest. N Engl J Med.

[4] Bernard SA, Gray TW, Buist MD, Jones BM, Silvester W, Gutteridge G (2002). Treatment of comatose survivors of out-of-hospital cardiac arrest with induced hypothermia. N Engl J Med.

[5] Nielsen N, Wetterslev J, Cronberg T, Erlinge D, Gasche Y, Hassager C (2013). Targeted temperature management at 33° C versus 36° C after cardiac arrest. N Engl J Med.

[6] Gajarski RJ, Smitko K, Despres R, Meden J, Hutton DW (2015). Cost-effectiveness analysis of alternative cooling strategies following cardiac arrest. Springerplus.

[7] Polderman KH (2009). Mechanisms of action, physiological effects, and complications of hypothermia. Crit Care Med.

[8] Reynolds JC, Frisch A, Rittenberger JC, Callaway CW (2013). Duration of resuscitation efforts and functional outcome after out-of-hospital cardiac arrest: when should we change to novel therapies?. Circulation.

[9] Kaneko T, Kasaoka S, Nakahara T, Sawano H, Tahara Y, Hase M (2015). Effectiveness of lower target temperature therapeutic hypothermia in post-cardiac arrest syndrome patients with a resuscitation interval of </=30 min. J Intensive Care.

[10] Seeger FH, Toenne M, Lehmann R, Ehrlich JR (2013). Simplistic approach to prognosis after cardiopulmonary resuscitation-value of pH and lactate. J Crit Care.

[11] Mullner M, Sterz F, Domanovits H, Behringer W, Binder M, Laggner AN (1997). The association between blood lactate concentration on admission, duration of cardiac arrest, and functional neurological recovery in patients resuscitated from ventricular fibrillation. Intensive Care Med.

[12] Kliegel A, Losert H, Sterz F, Holzer M, Zeiner A, Havel C (2004). Serial lactate determinations for prediction of outcome after cardiac arrest. Medicine (Baltimore).

[13] Grossestreuer AV, Abella BS, Leary M, Perman SM, Fuchs BD, Kolansky DM (2013). Time to awakening and neurologic outcome in therapeutic hypothermia-treated cardiac arrest patients. Resuscitation.

[14] Golan E, Barrett K, Alali AS, Duggal A, Jichici D, Pinto R (2014). Predicting neurologic outcome after targeted temperature management for cardiac arrest: systematic review and meta-analysis. Crit Care Med.

[15] Young GB (2009). Clinical practice. Neurologic prognosis after cardiac arrest. N Engl J Med.

[16] Oddo M, Rossetti AO (2014). Early multimodal outcome prediction after cardiac arrest in patients treated with hypothermia. Crit Care Med.

[17] Nishikimi M, Matsuda N, Matsui K, Takahashi K, Ejima T, Liu K, et al. CAST: a new score for early prediction of neurological outcomes after cardiac arrest before therapeutic hypothermia with high accuracy. Intensive Care Med. 2016;42(12):2106–7. 10.1007/s00134-016-4492-3 PMC5106489 27530297

[18] Torbey MT, Selim M, Knorr J, Bigelow C, Recht L (2000). Quantitative analysis of the loss of distinction between gray and white matter in comatose patients after cardiac arrest. Stroke.

[19] Metter RB, Rittenberger JC, Guyette FX, Callaway CW (2011). Association between a quantitative CT scan measure of brain edema and outcome after cardiac arrest. Resuscitation.

[20] Ajam K, Gold LS, Beck SS, Damon S, Phelps R, Rea TD (2011). Reliability of the cerebral performance category to classify neurological status among survivors of ventricular fibrillation arrest: a cohort study. Scand J Trauma Resusc Emerg Med.

[21] Hayakawa K, Tasaki O, Hamasaki T, Sakai T, Shiozaki T, Nakagawa Y (2011). Prognostic indicators and outcome prediction model for patients with return of spontaneous circulation from cardiopulmonary arrest: the utstein Osaka project. Resuscitation.

[22] Goto Y, Maeda T, Goto Y (2013). Decision-tree model for predicting outcomes after out-of-hospital cardiac arrest in the emergency department. Crit Care.

[23] Adrie C, Cariou A, Mourvillier B, Laurent I, Dabbane H, Hantala F (2006). Predicting survival with good neurological recovery at hospital admission after successful resuscitation of out-of-hospital cardiac arrest: the OHCA score. Eur Heart J.

[24] Rittenberger JC, Tisherman SA, Holm MB, Guyette FX, Callaway CW (2011). An early, novel illness severity score to predict outcome after cardiac arrest. Resuscitation.

[25] Ogura T, Nakamura Y, Nakano M, Izawa Y, Nakamura M, Fujizuka K (2014). Predicting the need for massive transfusion in trauma patients: the traumatic bleeding severity score. J Trauma Acute Care Surg.

[26] CAST score for iPad. https://geo.itunes.apple.com/jp/app/meidai-score-for-ipad/id1065338535?mt=8. Accessed 25 Oct 2016.

[27] CAST score for iPhone. https://geo.itunes.apple.com/jp/app/meidai-score-for-iphone/id1067612773?mt=8. Accessed 25 Oct 2016

[28] Nielsen N (2012). Predictive scores, friend or foe for the cardiac arrest patient. Resuscitation.

[29] Aschauer S, Dorffner G, Stertz F, Erdoqmus A, Laqqner A (2014). A prediction tool for initial out-of-hospital cardiac arrest survivors. Resuscitation.

[30] Becker LB, Aufderheide TP, Geocadin RG, Callaway CW, Lazar RM (2011). Primary outcomes for resuscitation science studies: a consensus statement from the American Heart Association. Circulation.

