# Supplementary figures and images for: A novel scoring system for predicting the neurologic prognosis prior to the initiation of induced hypothermia in cases of post-cardiac arrest syndrome: the CAST score
Source: Scand J Trauma Resusc Emerg Med. 2017 May 10;25:49. doi: 10.1186/s13049-017-0392-y (PMC5424379; doi:10.1186/s13049-017-0392-y)

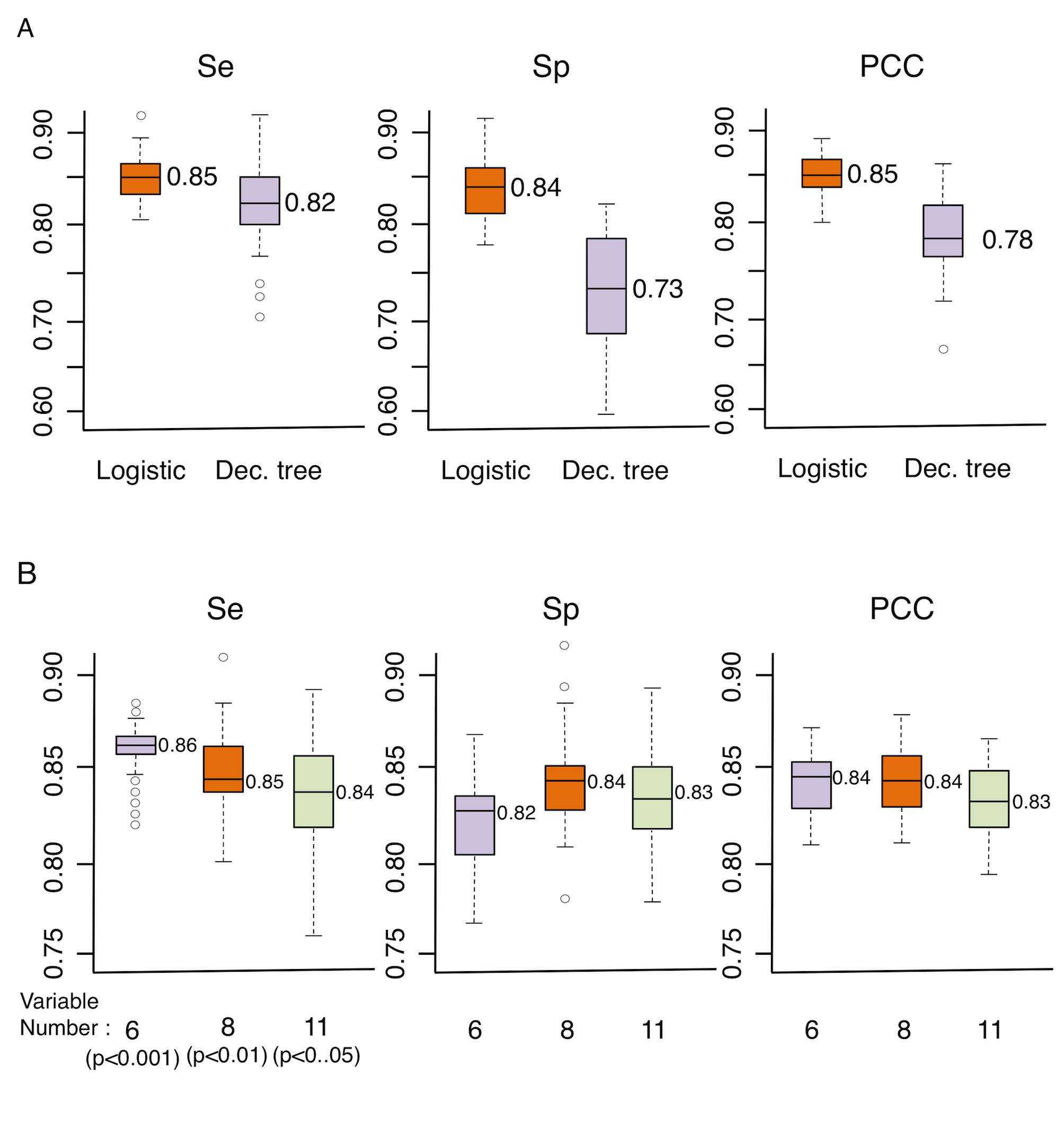

Supplement: Supplementary file 1 — Comparison of tentative scoring systems created using different algorithms and using different numbers of variables. The logistic regression algorithm yielded a higher sensitivity, specificity, and percentage of correct classification in the cross-validation study, than the decision-tree algorithm (A). The predictive accuracies of three scoring systems created using 6, 8, and 11 variables are shown (P values of 0.001, 0.01, and 0.05, respectively) (B). Logistic logistic regression algorithm, Dec. tree decision-tree algorithm, Se sensitivity, Sp specificity, PCC percentage of correct classification. (TIFF 16702 kb) [file 13049_2017_392_MOESM1_ESM.tiff]

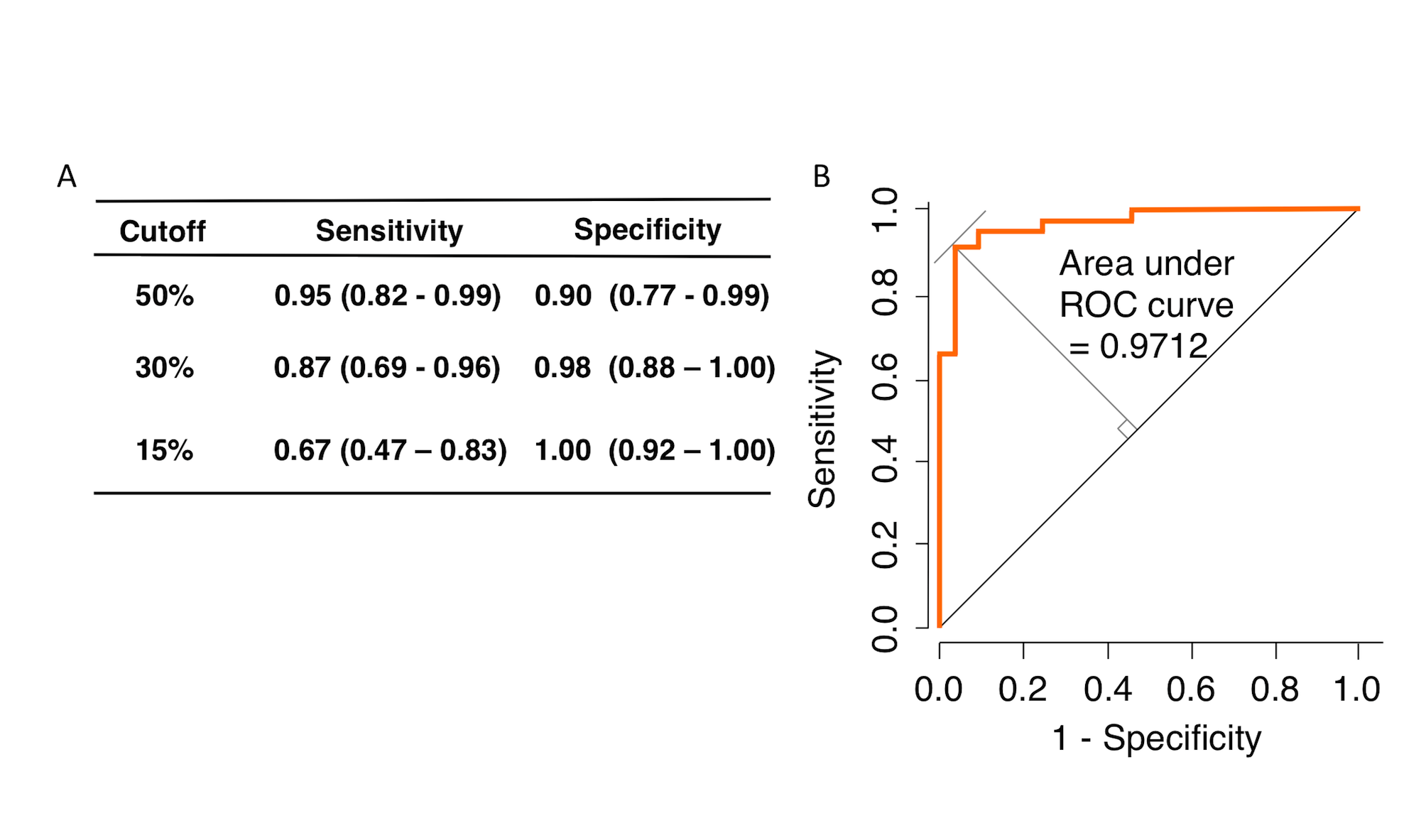

Supplement: Supplementary file 2 — Results of external validation of the tentative scoring system. The sensitivity, specificity, and percentage of correct classification for the external validation are shown (A). In these predictive accuracies, the cutoff values for discrimination between a poor and good prognosis was 50% (a “good prognosis” was identified if the probability of a good prognosis on the score was greater than 50%; otherwise, a “poor prognosis” was identified.), 30%, and 15%. The area under the receiver operating characteristic curve of the tentative score for which the logistic regression algorithm was applied with the different cutoff values (B). ROC receive operator characteristic. (TIFF 9281 kb) [file 13049_2017_392_MOESM2_ESM.tiff]
